# The impact of electronic records on patient safety: a qualitative study

**DOI:** 10.1186/s12911-016-0299-y

**Published:** 2016-06-04

**Authors:** Arabella Clarke, Joy Adamson, Ian Watt, Laura Sheard, Paul Cairns, John Wright

**Affiliations:** York Trials Unit, Department of Health Sciences, University of York, York, YO10 5DD UK; Department of Health Sciences, University of York, York, YO10 5DD UK; Bradford Institute for Health Research, Bradford Royal Infirmary, Duckworth Lane, BD9 6RJ Bradford, UK; Department of Computer Science, University of York, Yo10 5GH York, UK

**Keywords:** Qualitative, Electronic records, Patient safety, NHS, Maternity

## Abstract

**Background:**

Our aim was to explore NHS staff perceptions and experiences of the impact on patient safety of introducing a maternity system.

**Methods:**

Qualitative semi-structured interviews were conducted with 19 members of NHS staff who represented a variety of staff groups (doctors, midwives, health care assistants), staff grades (consultant and midwife grades) and wards within a maternity unit. Participants represented a single maternity unit at a NHS teaching hospital in the North of England. Interviews were conducted during the first 12 months of the system being implemented and were analysed thematically.

**Results:**

Participants perceived there to be an elevated risk to patient safety during the system’s implementation. The perceived risks were attributed to a range of social and technical factors. For example, poor system design and human error which resulted in an increased potential for missing information and inputting error.

**Conclusions:**

The first 12 months of introducing the maternity system was perceived to and in some cases had already caused actual risk to patient safety. Trusts throughout the NHS are facing increasing pressure to become paperless and should be aware of the  potential adverse impacts on patient safety that can occur when introducing electronic systems. Given the potential for increased risk identified, recommendations for further research and for NHS trusts introducing electronic systems are proposed.

**Electronic supplementary material:**

The online version of this article (doi:10.1186/s12911-016-0299-y) contains supplementary material, which is available to authorized users.

## Background

Electronic records are being introduced into many healthcare organisations around the world [1] and contain patient information; personal, diagnosis, condition and treatment details [2]. In the UK, electronic records are seen as one mechanism by which the NHS can become safer and more efficient. For example, NHS IT policy claims that electronic records have the potential to ‘improve health and transform the quality and cost of healthcare’ [3]. The goal of ‘electronic records for all’ was proposed in 1998 by the NHS IT strategy ‘Information for Health’ [2] but remains an ambition of the NHS, with recent IT strategies (2013, 2014) calling for a paperless NHS by 2020 [3, 4]. However, despite an estimated £10 billion being invested since 2002 [4, 5], progress has been slow.

Policy and financial support for NHS trusts to implement electronic records implies a strong evidence base supporting the idea that these systems can improve health outcomes and quality of care. In reality, the literature is limited, as demonstrated in a recent systematic review [6] of eHealth technologies and their impact on the quality and safety of healthcare, which concluded that there is a gap between the proposed and empirically evidenced benefits of eHealth technologies. In addition, there is little consideration given in existing literature to potential negative effects of these systems on patient safety, with existing evidence under-cited and predominately from the U.S whose health service has different economic, organisational and structural foundations from the UK [7–11]. In their review [6], Black et al provided some discussion into this and suggested that the lack of evidence may be due to publication bias, with potential conflicts of interest making it particularly difficult to publish negative findings [6].

Given policy pressures on hospitals to implement electronic records it is crucial that potential risks and safety implications are examined, not just potential benefits. NHS IT policy is often criticised for not evidencing its aims, however to do so, evaluations of not only the positive, but negative impacts of implementing electronic records are essential [12]. In the NHS there is not a good culture of error reporting as it often leads to blame allocation rather than process improvement. Risks are under-reported [13] and so exploring perceptions and experiences was considered a good way of understanding current risks to patient safety, as opposed to incident reports or observations. This study explored perceptions and experiences of staff regarding the impact upon patient safety of implementing a maternity system. Implementing an electronic system into a maternity unit differs from other specialties, as since the introduction of the ‘co-operation card’ in 1956, which made paper hand-held records integral to maternity shared care [14], women in the UK have been responsible for their own records throughout pregnancy.

## Methods

The study explored the implementation of a maternity system in a Women’s and Newborn unit in a teaching hospital in the North of England. The trust offers care to approximately 6,000 women and families annually. To protect the anonymity and confidentiality of the participating trust and the maternity system’s supplier it has not been named and is referred to as ‘the system’ hereafter. The system which can be integrated into a broader hospital wide Electronic Patient Record has been implemented as a departmental system to allow obstetric journeys to be electronically recorded. When fully implemented, it will be used for a range of care activities: antenatal, delivery, postnatal and community. Data collection took place between April and November (2014) during the first year of the system's implementation. Ethical approval was obtained by the University of York Health Sciences Research Governance Committee in January 2014. NHS R&D approval from the study site was also obtained in April 2014.

### Theoretical approach

We drew upon socio-technical thinking [15], which aims to determine how social influences affect the performance of technical systems [16]. This approach challenges the idea that IT projects fail due to technological reasons alone [17] by giving equal weight to social and technical issues affecting the implementation and adoption of technology in healthcare [16]. Socio-technical thinking has been applied to studies evaluating the implementation of electronic records [18] based on the premise that these systems do not merely store information, but influence care. The approach assumes ‘people and technologies are linked within complex, dynamic, socio-technical networks that enable and inhibit what is possible within certain situations and contexts’ [18]. This study used socio-technical thinking when analysing interview data to establish themes illustrating clinician’s perceptions and experiences of how the system has impacted patient safety [19].

### Recruitment and sampling strategy

As the system was implemented in stages and interviews were conducted during the first year of the system's implementation, the amount of time that staff had been using the system when interviewed varied. It was anticipated that staff and wards across the maternity unit would be using the system differently because of their varying roles and responsibilities. To try and reflect this variation, a purposive sampling frame was used to recruit from a range of staff groups and grades to understand their perceptions and experiences of the system’s impact on patient safety across a range of usage. Staff from the unit who were directly involved in the implementation of the system were recruited as key informants, their perceptions and experiences of the system potentially differing from those not actively involved in supporting the system’s implementation.

Participants were recruited via telephone, email and a junior doctors’ WhatsApp group. The purposive sampling frame was used until a sample that qualitatively represented a range of specialities and professions throughout the maternity unit was achieved.

### Participants

Of the 29 members of staff invited to take part in the study, 19 individuals consented and were interviewed. The sample comprised 11 midwives (grade 5 to 7), 7 doctors (Senior House Officers to Consultant) and 1 health care assistant. Participants were recruited from a range of different wards throughout the maternity unit including; maternity assessment centre, community, birth centre, labour and the antenatal day unit and had between 5 and 28 years professional experience. 4 participants were members of the system support team whose role was to champion the system and assist staff users.

### Interview design and content

Interviews were conducted face-to-face, were semi-structured and followed a topic guide (Additional file 1) which was informed by the literature and prior discussion with key informants. Findings presented here are part of a wider study and so following informed consent, interviewees were asked about their perceptions and experiences of the benefits and barriers to the implementation of the system as well as the impact of the system upon their practice and patient safety.

### Analysis

Interviews were transcribed verbatim and analysed thematically in five stages: transcription, familiarisation, coding, theme development and data reporting [20]. The research team were consulted throughout code and theme development. Following each interview, reflexive notes [21] were taken with personal and methodological changes or challenges noted and considered during analysis.

## Results

During interviews, NHS staff described how they perceived, and in some instances experienced, an increased risk to patient safety. Significantly more challenges were reported (and with more emotion) than benefits of the system. A concerted effort to maintain balance was made during interviews; however it became clear that challenges to patient safety outweighed perceived benefits and so these concerns were allowed to emerge unconstrained by the interviewer. From the analysis the two key themes emerging for perceived increased risk related to social (e.g. computer literacy) and technical factors (e.g. system design).

### Social factors affecting the safe use of the system

The introduction of the system was perceived to be associated with an increased potential for inputting error, as staff were not used to using the system and so felt they were more prone to making mistakes. Reflecting this belief, inputting errors were considered most likely to occur when junior doctors or new staff joined the wards and/or following upgrades to the system, which required an element of re-learning. Interviewees with low levels of computer literacy were concerned that their lack of typing skills could make them more prone to inputting errors. For some, lack of confidence and nervousness at the prospect of typing in front of colleagues and patients caused them to feel scared to use the system. One participant described how this had resulted in some staff shying away from using the system altogether, placing their computing workload onto others. Concerns over inputting errors were exacerbated by the potential implications that entering incorrect information onto the system may have for patient safety. For example, inputting errors can make it appear that a patient has received care or has a condition they do not have, both of which could impact upon the length of stay and treatment provided. Additionally, if inputting errors are not noticed by staff and incorrect information remains on the system, there is the potential for legal as well as medical consequences:

*Midwife 062712*: *it’s my legal documents and I’m not the quickest typist in the world, I’m not a trained typist, so the amount of time it took me to make sure that you’ve got everything spelt right and…written down because our documents follow us for 25 years (41-44).*

Some participants were concerned that less detail is being entered onto the system than paper records allowed. This was largely attributed to typing feeling more disjointed and taking longer than writing. To save time some staff only answered mandatory questions on the system, which would not cover everything necessary for all patients and all staff, increasing the risk that information may go unrecorded. The accuracy of patient records was also questioned. Although, there is a risk that incorrect information could also have been written into paper records, interviewees felt that the system brought new risks, from staff using the system differently and the system allowing the same information to be inputted into different places; increasing the risk of missing information. Participants described how mistakes were most likely when first using the system and that they have to trust that their colleagues are inputting information correctly and in the right place. Further concerns that staff are not highlighting risk factors, allergies and test results adequately were raised:

*Consultant 180703: in the olden days if somebody had a full blood count on their notes was a little box to say they had it done so if I saw patient and did a full blood count there would be a little box and hopefully that would prompt the next person when they see the patient to say ‘she has a box from last time so let me check that full blood count’ whereas I am not sure we are highlighting that adequately in the way we are using it (158–162).*

### Technical factors affecting the safe use of the system

Compared with paper notes, staff reported finding it harder to find the information they need. This was attributed to the design of the system making it difficult to use and navigate around. Consequently, participants perceived there to be an increased risk that patient information may be missed, with this risk elevated when the system was first implemented and staff were becoming accustomed to using it and how information was presented. The way the system presents information was a particular issue for women classed as frequent attenders, who have a large number of record entries. For these women, because the system presents information chronologically, important information can become buried under large amounts of routine information. Although this could be argued to be an issue with paper records, clinicians were used to paper notes and so were able to quickly find the information they needed. To avoid missing information when using the system some clinicians defaulted to asking women to tell them of important clinical information. This was not always possible, for instance, in emergency situations and where English is not the patients’ first language. Failing to identify important information in patient notes could have significant implications for safety:

*Midwife 133002: I think we will miss something because we don’t know where to look for the information or we’ll miss a problem and an alert and it will lead to a baby becoming septic or a mother becoming unwell (107–108).*

The lack of flexible data entry methods such as being able to draw diagrams was also criticised as staff are not able to elaborate their typed data entry particularly when documenting operative procedures. Consequently, patient information is being presented in a standardised fashion which although desirable, was felt in this instance to result in missing the nuances and details of individual patients and procedures. The implications for not having sufficient information, particularly relating to patient histories, allergies and risk factors were discussed:

*Consultant 042202: people were picking up from the down select button they were going to the minimum, easiest quickest route so every operations looked exactly the same…all caesarean sections looked exactly the same…and as a clinician for 15–20 years I know that not every caesarean section is the same (169–171)*

Staff also explained how they have to ‘fight over’ insufficient numbers of computers. Time spent waiting for a computer to become available and then logging onto the system was perceived to be increasing the length of discharge and clinic waiting times. Furthermore, computers that are available are placed at the opposite ends of the ward to women, forcing staff to leave women to access the system; a problem exacerbated by the lack of handheld devices. Participants described how this is a particular issue in emergency situations where they are faced with the conundrum of either leaving women and risking them deteriorating as they try to locate and access an available computer, or staying to treat women without having ready access to their records. The implications of not having access to patient records were discussed, particularly in situations where women cannot speak English and so cannot communicate key information relating to their previous history:

*Midwife 091203: my main concern would be that we would miss women that come in and whether they are MRSA positive and it’s stuck to the front of the notes and it won’t be any more… simple things like that to a HIV positive and we are not going to know that until we get to a computer and we might not have time to get to a computer which…could really affect the delivery… of the baby so that would be the biggest issue is not knowing if they come in and they do it quickly not being able to access the computer its potentially putting them at risk and we can be putting us at risk (87–95).*

Technical issues also caused problems with access to information. Staff are required to log in and out of the system individually for each woman and are not allocated their own clinic rooms. Clinicians are subsequently finding it difficult to change rooms and log into the system within the 5 minutes allocated for clinic appointments, causing delays. Participants explained how particularly during busy clinics when staff are repeatedly logging in and out of the system, on the same computer, the system is “freezing” and sometimes “crashing”. This has caused access to patient records to be suspended and in some cases women to be sent home from clinics. The implications of this were seen to be exacerbated by staff in these situations resorting to ad-hoc paper documentation, increasing the risk of information being lost or not being inputted once the system is back up and running. Staff were particularly concerned and unclear as to who is liable in the event of a patient safety incident occurring as a result of the system crashing and access to records being suspended. Community midwives shared this anxiety surrounding liability following ‘near misses’ in the community where the system could not be accessed due to poor internet access.

*Midwife 051602: I’ve recently had a case where I couldn’t get a signal….and …this was a very high risk pregnancy, I did know the patient very well…the high risk issue poses a risk to us as well as to her and if I’d have gone out and say she had delivered at home unprepared….and no access to any records I think is a massive risk to this organisation and to the midwives and the woman, there was no signal…it was in the city center…I’m quite scared by it to be honest (55–60).*

Participants also described how the quality of care has been affected by clinicians not having access to patient information before consultations. Direct comparisons were made with the paper records, which allowed clinician’s to ‘flick through’ patient’s notes beforehand. However, with all patient information being on the system, clinical staff felt they were having to enter consultations ‘blind’ without any understanding of the patients history or reason for attending clinics necessitating obtaining this information through small talk; which was reported as challenging given the time constraints of appointments. One participant described how not having access to patient notes prior to appointments has led to some ‘real faux-pas’:

*Doctor 111609: It would be nice to look at somebody’s notes on a laptop or something outside the room and see what you are talking about rather than go into a room cold with a patient and then say ‘who are you? Why are you here? How many times have you been pregnant?’ and they say ‘three times’ and you say ‘oh how are your three children?’ and they go ‘well actually one of them died’ (154–160).*

The system’s data entry methods which mainly involve the use of tick boxes and drop down menus were thought to elevate the risk of inputting errors. Particularly under time constraints it was considered too easy for clinicians to tick the wrong box. Midwives described how this had led to incorrect results or conditions for women being entered which if undetected can impact upon women’s treatment and length of stay. Additional implications from inputting errors are discussed below:

*Midwife 081203: the implications could be the wrong information is down, the wrong date of birth, or the wrong NHS number, It can cause problems further down the line and I guess the problem is that from a midwife point of view people might not actually be aware of what those implications might be if the baby doesn’t have an NHS number or it hasn’t been registered properly they turn up to the registrar’s office to get a birth certificate and they’ve put the wrong gender down. I mean that’s quite a common mistake that people make and it’s not because they don’t know if it’s a boy or a girl it’s just they’re tired or the cursor just flips from female to male or there’s contradictory information (151–159).*

Lastly, participants felt that the potential impact of errors made on the system is elevated by staff’s inability to rectify their mistakes as they only have the ability to input but not edit records. Staff must rely upon a support team or colleagues who have been made “super users” to correct errors. Additionally, the number of clinicians who have received the extended training and have been made “super users” is limited and the support team are only available during office hours Monday-Friday. Therefore, should an error be made on a Friday at 6.00pm this incorrect information would remain on a woman’s record potentially until after the weekend, unless a super-user was available. One example of the implications of this is outlined below:

*Midwife 091203: it wouldn’t let us save this new baby because it didn’t believe that she’d had another pregnancy and it wouldn’t not let us do it at all and we’d tried all sorts but this was the documentation for the parents to take home that I just couldn’t give them because the system wouldn’t let me finish it and that was on the Saturday and nobody was in till the Tuesday (183–186).*

## Discussion

Interviews revealed that NHS staff perceived there to be an increased risk to patient safety during the first 12 months of the system’s implementation. Some staff were able to give specific examples of where they thought use of the system had put patient safety at risk. The social and technical factors identified here, were largely a result of human factors and system design which were felt to have increased the risk of inputting errors and of missing patient information.

Our study has identified perceived constraints and limitations of new electronic records. It is surprising that there has been so little research into the potential harms of implementing electronic systems into the NHS on patient safety. Previous research has focused on potential benefits of these systems, such as reducing inputting errors and adverse drug events [22–26]. This study’s findings correspond with a limited and predominately U.S evidence base, which has identified human errors and technical issues associated with Health Information Technology [8–12, 27–33] and which have the potential to increase the risks for patient safety. Sittig and Singh’s [10] framework for the development of electronic record specific patient safety goals can be used to help conceptualise the findings within this study and those within the existing literature. The framework, suggests a 3-phase approach for measuring and monitoring safety concerns, categorising concerns as those which are specific to technology (e.g., issues with the system crashing or an insufficient number of computers available), or which result from the incorrect use of technology (e.g., inputting error due to poor computer literacy). In its final phase, the framework considers the use of technology to monitor risks, healthcare processes and outcomes for identifying concerns before a patient is harmed. The framework may therefore provide a useful mechanism for raising awareness of the potential risks associated with electronic records. The third phase of the framework may also prove useful for highlighting the limited evidence surrounding the use of technology to monitor patient safety risks.

The study adds to an emerging but limited evidence base that has reported potential negative impacts of electronic systems upon patient safety. The purposive sampling frame enabled a more comprehensive representation of the way that the system is perceived and experienced to of impacted patient safety to emerge. Additionally, the use of socio-technical thinking during the interpretation of the study’s findings allowed a more in-depth interpretation of the data that went beyond a descriptive list of themes to be obtained. The study’s main limitation was that it was undertaken with NHS staff within a single maternity unit at a NHS trust. A number of the issues identified within this study were the result of the particular systems’ design and implementation. For instance, the system did not have the capacity for diagrams and there were insufficient computers stationed in inappropriate places. These issues, that had potential ramifications for patient safety, may not necessarily be experienced by other trusts implementing different electronic systems. However, it is likely that many of these factors would be transferable to other similar large hospital trusts and other clinical specialties.

It has been suggested that to effectively evaluate technology and its impact on safety, evaluation needs to occur continuously prior to, during and following implementation [34]. In this study, interviews took place at just one point in time-during the first year of the system’s implementation’. It is therefore possible that participants’ views may have changed or be representative of only that stage of implementation. Although it would have been preferable to conduct interviews throughout the ‘evaluation lifecycle’ [34] this was not possible due to significant delays to the system’s implementation restricting the time available for interviews to be conducted.

Policymakers around the world are placing hospitals under increasing pressure to implement electronic systems, through funding [4] and policies emphasising the potential of these systems to ‘transform healthcare’ and improve patient safety [3]. The absence of any consideration of potential negative impacts of introducing electronic records in policy, particularly in the early stages of implementation, could result in unrealistic expectations and patient safety being jeopardised. On the basis of the study’s findings, a number of recommendations would seem worthy of consideration (Table 1).

**Table 1:** Recommendations for implementing technology into healthcare organisations

Recommendations	
Engagement of front line staff and feedback	Hospitals should not underestimate and should acknowledge the potential risks by providing regular opportunities for front line staff to voice their concerns. Trusts should also work closely with clinicians to increase their vigilance and preparedness for potential errors in the early phases of implementation.
Technology and hardware	Prior to introducing systems, hospitals should ensure that sufficient hardware is placed in appropriate locations to prevent delays to clinics and risks in emergency situations. Where technologically and financially possible, mobile devices or computers on wheels may help to alleviate situations where staff are choosing between accessing information and staying with acutely unwell patients. During system down times or failures, back up or well communicated procedures and policies should be incorporated and understood by all staff. For instance, if paper is to be reverted to then it should be a mandatory requirement that any information recorded on paper during these periods should then be inserted onto the system once it is ‘up and running’ to prevent important information from being lost or missed in future.
Training and support	Training should be provided on an on-going basis so that those finding using the system difficult can gain extra support with basic IT training sessions advisable. IT support teams should be available 24/7 to reflect the 24/7 provision of clinical care in hospitals. To ensure that those with poor computer literacy are supported, basic IT training, including typing skills should be made available, particularly during initial implementation. Hospitals should also be responsible for ensuring that all staff are computer literate.

## Conclusions

This study identified that during the first year of implementation there may be a period of increased risk to patient safety, as staff become accustomed to using the system. Given the global focus on digitising health, it is important that organisations are aware of and do not underestimate the potential risks. This study has identified perceptions and so further research is needed to determine the actual level and scale of the risk during early implementation of electronic systems. This could be achieved by quantifying errors and harm using robust case note review or through linking qualitative findings around increased risk with standardised hospital reporting procedures such as incident reports which would help to ascertain and validate perceptions and experiences of risk. Additionally, research that seeks to determine the impact upon patient safety during initial implementation should aim to study a number of different electronic systems across different trusts to identify common risk factors.

## Abbreviations

IT, Information Technology; NHS, National Health Service; UK, United Kingdom; US, United States.

## Additional file

Additional file 1: Topic guide for interviews with NHS staff. Topic guide used for the qualitative interviews with study participants. Within the main text the topic guide is referenced as Additional file 1. (DOCX 18 kb)
