# Supplementary material for: The impact of electronic records on patient safety: a qualitative study
Source: BMC Med Inform Decis Mak. 2016 Jun 4;16:62. doi: 10.1186/s12911-016-0299-y (PMC4893301; doi:10.1186/s12911-016-0299-y)
Supplement: Additional file 1: — Topic guide for interviews with NHS staff. Topic guide used for the qualitative interviews with study participants. Within the main text the topic guide is referenced as Additional file 1. (DOCX 18 kb) [file 12911_2016_299_MOESM1_ESM.docx]

## Additional File 1: Topic guide for interviews with NHS staff

**At the beginning of all interviews:**

- Introduce self
- Introduce study
- About audio recording/anonymity/confidentiality
- Explain how data will be used
- Obtain Verbal Consent

1. Background information (how long have you been in your specialty, years’ experience etc.)
2. Were you made aware or are you aware of the purpose of the technology?
   1. What is the purpose of the technology to you?
3. When the technology was introduced what benefits did you think would result?

- Who did you think the technology would benefit most?

1. When the technology was first introduced how did you feel?
   1. Is it a good idea?
   2. Did you or do you still have any concerns?
   3. And what about now?
2. Can you tell me how you were introduced to Eclipse (any training etc)

- Did you have any training, how did you feel about the training
- How much time did you have to invest in the new system how did this make you feel?

1. How did you think the introduction of the technology would impact upon your practice?
   1. Will it help or impede you
   2. How has it affected your practice
2. How compatible was the new technology to your previous methods?
3. What benefits have you seen to using the new system?
4. Have there been any barriers to using the system
5. Have you experienced any disadvantages to using the system?
6. Have you seen any patient safety related impact both positive or negative?
7. How do you feel the patients have responded to the technology?
   1. Do you think it has it affected your interaction with them?

**End the interview:**

- Thank participant ask if they have any other comments
- Explain again about how data will be used and reiterate about anonymity and confidentiality
- Provide opportunity for questions and states that the chief investigator is contactable after the interview should questions arise.
- Ask the participant if they would like to receive a summary of the results of the study.
